# Recent advances in biomedical applications of lyotropic liquid crystals

**DOI:** 10.1016/j.apsb.2025.12.008

**Published:** 2025-12-08

**Authors:** Guojin Liu, Yuanmei Yang, Qing Wu, Zhongjian Chen, Nadia M. Hamdy, Amr Amin, Gang Chen, Yi Lu

**Affiliations:** a Guangdong Provincial Key Laboratory of Pharmaceutical Preparations Research and Evaluation, School of Pharmacy, Guangdong Pharmaceutical University, Guangzhou 510006, China; b Key Laboratory of Smart Drug Delivery of MOE, School of Pharmaceutical Sciences, Fudan University, Shanghai 201203, China; c National Key Laboratory of Advanced Drug Formulations for Overcoming Delivery Barriers, School of Pharmaceutical Sciences, Fudan University, Shanghai 201203, China; d Department of Stomatology, Shanghai Fifth People’s Hospital, Fudan University, Shanghai 200240, China; e Shanghai Skin Disease Hospital, Tongji University School of Medicine, Shanghai 200433, China; f Biochemistry Department, Faculty of Pharmacy, Ain Shams University, Cairo 11566, Egypt; g College of Medicine, University of Sharjah, Sharjah 27272, United Arab Emirates; h Faculty of Science, Cairo University, Cairo 12613, Egypt; i The University of Chicago, Chicago, IL 60637, USA; j Fudan Zhangjiang Institute, Shanghai 201203, China

**Keywords:** Lyotropic liquid crystals, Lamellar, Cubic, Hexagonal, Nanoparticles, Gels, Long-acting therapy, Biomedicine

## Abstract

Lyotropic liquid crystals (LLCs), formed through the self-assembly of amphiphilic molecules in polar solvents, offer thermodynamic stability and tunable mesophases (lamellar, hexagonal, and cubic), making them a versatile platform for biomedical applications. Their structural adaptability enables enhanced drug stability, improved bioavailability, and controlled release, which are advantageous for various therapeutic strategies. LLC-based systems have shown significant promise in drug delivery, long-acting therapies, anti-infective treatments, and wound healing. Their diverse formulation options, including gels, nanoparticles, and *in situ* forming precursors, support multiple routes of administration, such as oral, intravenous, dermal, ocular, and intranasal. This review summarizes recent advances in the design and functionalization of LLC systems, with a focus on their ability to overcome physiological barriers, enhance therapeutic efficacy, enable targeted delivery, and support prolonged treatment regimens. Challenges in clinical translation and future research directions are also discussed to facilitate the transition from bench to bedside.

## Graphical abstract

This review provides a comprehensive overview of recent advancements in biomedical applications of lyotropic liquid crystals, particularly in overcoming physiological barriers, site-specific delivery, long-acting therapy, and promoting wound healing.

**Figure:**
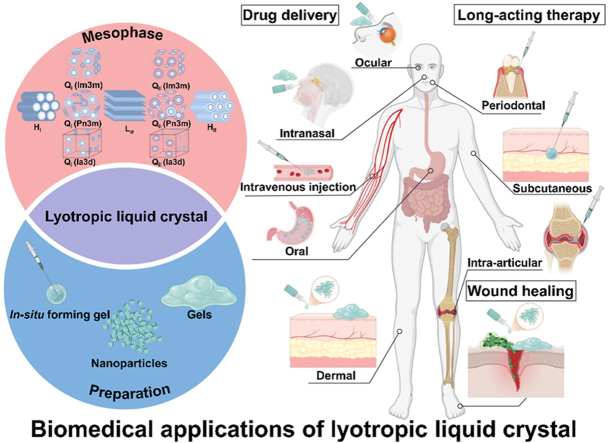

## 1 Introduction

Liquid crystals (LCs) are a distinct state of matter exhibiting both fluidity and molecular order, which are characteristics of liquids and crystalline solids, respectively^1^. The phenomenon was first observed by Friedrich Reinitzer in 1888 in cholesteryl benzoate and was later termed “liquid crystals” by Otto Lehmann in 1904^2^. Foundational studies in the 1930s‒1960s, including McBain’s work on myelin figures and Vittorio Luzzati’s X-ray analyses, established the structural basis of surfactant–water self-assemblies, such as lamellar, hexagonal, and cubic phases^3^. Further theoretical developments in the 1970s‒1980s included Jacob Israelachvili’s Critical Packing Parameter (CPP) theory, which clarified the relationship between molecular geometry and mesophase behavior^4^; meanwhile, Kåre Larsson pioneered the use of glycerol monooleate (GMO) in food and cosmetic applications^5^. In the 1990s and 2000s, LCs gained prominence in biotechnology, notably in the crystallization of membrane proteins, as demonstrated by Martin Caffrey’s work, which contributed to the 2012 Nobel Prize in Chemistry. Naturally occurring LCs are found in key biological structures such as cell membranes, myelin sheaths, arteries, and chloroplasts, where their dynamic, self-assembling properties support essential functions, including structural organization and signal transduction^6^. These properties make LCs especially attractive for biomedical applications, such as biosensing, targeted drug delivery, and biomimetic material development.

**Figure 1:**
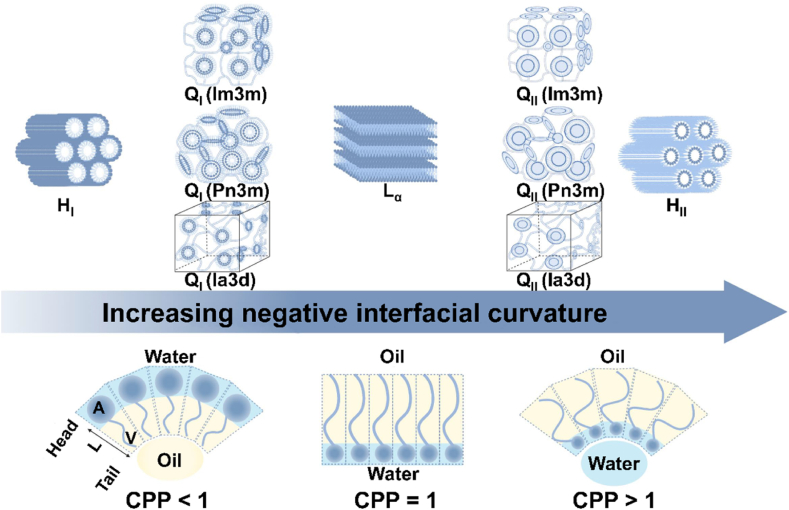
The mesophases of lyotropic liquid crystals. Created with BioRender.com.

LCs are mainly divided into thermotropic (TLCs) and lyotropic (LLCs) types^7^. TLCs, which form through temperature-induced phase transitions in pure compounds or mixtures, have limited biomedical utility due to issues such as toxicity, instability, and complex fabrication^8^. In contrast, LLCs are formed through the self-assembly of amphiphilic molecules in polar solvents, yielding thermodynamically stable structures^9^. Depending on the spatial arrangement of the amphiphiles, LLCs can adopt lamellar (L*α*), hexagonal (H), or cubic (Q) mesophases (Fig. 1)^10^. The lamellar phase consists of planar lipid bilayers arranged in a one-dimensional lattice interspersed with water layers and exhibits zero curvature, resembling biological membranes. The hexagonal phase comprises rod-like micelles organized in a hexagonally packed array. The cubic phase features a three-dimensional bicontinuous network that separates two distinct, non-intersecting aqueous channels. Cubic phases are further categorized by their symmetry groups: primitive (P, Im3m), diamond (D, Pm3m), and gyroid (G, Ia3d). Subscripts I and II denote normal (*e.g.*, H_I_, Q_I_) and inverse (*e.g.*, H_II_, Q_II_) phases, respectively, based on the orientation of the hydrophilic and hydrophobic domains.

The mesophase transitions are governed by CPP^10^, which predicts the type of self-assembled structure based on molecular geometry. CPP is defined as the ratio of the hydrophobic chain volume (V) to the product of the hydrophilic headgroup area (A) and the hydrophobic chain length (L) (Fig. 1). A CPP value < 1 suggests a normal phase (*e.g.*, H_I_ and Q_I_) where the hydrophilic head group area is larger than the hydrophobic tail area. Conversely, a CPP value > 1 indicates a reverse phase (*e.g.*, H_II_ and Q_II_) where the hydrophobic tail area is larger. A CPP value equal to 1 indicates the comparable head group and tail areas, and thus, cylindrical-shaped molecules tend to form lamellar phases. CPP is not a fixed property and can be influenced by various factors such as amphiphile concentration, temperature, solvent polarity, and the presence of additives. These parameters can drive transitions between different mesophases. For example, in the GMO/water system, both water content and temperature determine the type of liquid crystal formed^11^. At 37 °C and 5% water content, GMO molecules self-assemble into a lamellar phase. As water content increases to 20%‒40%, the hydrophilic headgroups orient toward the aqueous environment while the hydrophobic tails aggregate inward, forming spherical micelles that organize into a cubic phase. Beyond 40% water content, the cubic phase coexists with excess water. Increasing the temperature further induces structural transitions; at 100 °C, reverse micelle formation is observed. When additives are introduced into the GMO/water system, they can interact with the GMO headgroups, altering the packing parameter and consequently changing the liquid crystal type^12^. Lipophilic additives increase the hydrophobic volume, raising the CPP and shifting the structure from cubic to hexagonal. In contrast, **hydrophilic additives** expand the headgroup area, lowering the CPP and promoting a transition from cubic to lamellar phases through interactions with the polar headgroups of GMO.

LLCs are widely used in biomedicine, with common amphiphilic lipids like GMO and phytantriol (PT) forming the foundation for LLCs^13^^,^^14^. When dispersed in water, these amphiphiles form semi-solid, viscoelastic gels composed of a three-dimensional nanostructured network that entraps water, resulting in the formation of LLC gels^15^. The mesophases can be exploited to prepare liquid crystalline nanoparticles (LCNPs)^16^^,^^17^. Cubosomes and hexosomes are typical LCNPs where the lipidic molecules are arranged in cubic and hexagonal phases, respectively^18^. LLCs are widely studied for drug delivery applications due to their capacity to encapsulate both hydrophilic and hydrophobic agents, enhance the solubility of poorly water-soluble drugs, and modulate drug release profiles^19^. Their internal nanostructure enables precise control over drug diffusion, reducing systemic side effects while enhancing therapeutic efficacy^20^. Importantly, LLCs exhibit excellent biocompatibility and biodegradability, as their constituent lipids, such as GMO and PT, are metabolized by endogenous lipases into glycerol and fatty acids, which are naturally eliminated through biological pathways. This favorable safety profile is supported by studies reporting high cell viability (>90%)^21^ and minimal inflammation at therapeutic doses^22^. The biocompatible and biodegradable nature of lipids used in LLCs makes them highly suitable for a range of biomedical applications.

This review highlights advances in LLC-based biomedical applications over the past five years, with a particular focus on drug delivery *via* multiple administration routes, including oral, intravenous, transdermal, ocular, intranasal, and local implantation. Oral and intravenous delivery were among the earliest applications of LLCs, while transdermal systems have more recently garnered attention for their ability to enhance percutaneous absorption and promote wound healing through the localized delivery of antibacterial, angiogenic, and anti-inflammatory agents. Emerging applications include ocular, intranasal, and implantable formulations, which offer prolonged local retention, sustained release, and improved penetration of biological barriers, thereby enhancing therapeutic outcomes. This review systematically examines LLC development across these administration routes. It summarizes the composition, formulation types, physicochemical properties, and therapeutic achievements of various LLC systems in tabulated form. Additionally, it discusses mechanisms of action and key factors influencing LLC performance. For foundational knowledge on LLCs, readers are referred to recent comprehensive reviews^23^, ^24^, ^25^.

## 2 Oral administration

Oral administration is one of the most widely preferred routes for drug delivery owing to its convenience, non-invasiveness, and patient compliance^26^. However, one of the primary challenges for several drugs, particularly those classified as Biopharmaceutical Classification System (BCS) Class IV, is their poor solubility and low bioavailability. To address these issues, novel drug-delivery systems are being developed, with LLCs offering a promising solution.

The enhanced oral bioavailability from LLCs is related to the digestion and absorption of lipids^27^. The ingestion of lipids stimulates the secretion of pancreatin, bile salts, and phospholipids, leading to the digestion of lipids^28^^,^^29^. The digestion products, such as diglyceride, monoglyceride, and fatty acids, are formed on the surface of a lipid droplet, where they assemble into different types of LCs, depending on the chain length of the lipids^30^. The dilution of the liquid crystalline phases results in a colloidal phase. Poorly water-soluble drugs are solubilized in these secondary phases and are transported across the unstirred water layer for absorption. LLCs are biomimetic drug-delivery systems. In addition to solubilizing lipophilic drugs, they can incorporate hydrophilic drugs and protect them against gastrointestinal degradation^25^.

Cubosomes enhanced the oral bioavailability of both lipophilic and hydrophilic drugs (Table 1 31, 32, 33, 34, 35). Both carvedilol and coenzyme Q10 are BCS II-type drugs with poor solubility but good permeability. Their oral bioavailability is mainly limited by their poor solubility. The cubic phase not only solubilized the two drugs but also enabled a sustained release lasting 24 to 48 h^31^^,^^32^. Consequently, the nanoparticles persisted the carvedilol’s reduction effect in mean arterial pressure for up to 24 h and enhanced the coenzyme Q10’s hepatoprotective effect. Cromolyn sodium is highly hydrophilic and poorly permeable to the gastrointestinal epithelia, exhibiting poor bioavailability (approximately 1%). The cubic phase could accommodate cromolyn sodium in the water channel and induce a sustained release (70%‒80% drug release in 24 h)^33^. More importantly, the cubosomes increased the permeability of cromolyn sodium by 1.2- to 20-fold. Finally, a 1.7- to 4.2-fold increase in absolute bioavailability was achieved over the solution, depending on the weight ratios of the compositions.

**Table 1:** Advances of LLCs in oral drug delivery.

Drug	Disease	Material	Phase	Properties	Achievement	Ref.
Carvedilol	Hypertension	GMO; P407; polyvinyl alcohol;	Q_II_	Size: 141 nm; PDI: 0.166; Zeta potential: 4.9 mV	An *in vitro* sustained release behavior; comparable *in vivo* antihypertensive performance that was maintained for 24 h.	31
		GMO; P407; cremophor RH 40		Size: 105 nm; PDI: 0.247; Zeta potential: 6.3 mV		
Coenzyme Q10	Hepatoprotective effect	GMO; P407	Q_II_	Size: 132.4‒223.2 nm; Zeta potential: <−21.3 mV; EE: 44.69%‒75.96%	Release of coenzyme Q10 was sustained to 48 h. An improved hepatoprotective effect compared to plain drugs.	32
Cromolyn sodium	Allergic conditions	GMO; P407	Q_II_	Size: 133.5 nm; PDI: 0.21; Zeta potential: −37.5 mV; EE: 59.49%	1.2–20 fold increase in permeability and 1.7–4.2 fold higher absolute bioavailability than the solution, depending on the weight ratios of the compositions.	33
Pexiganan	*Helicobacter pylori* infection.	Phosphatidylcholine; GDO; *N*-methyl-2-pyrrolidone	H_II_	*In situ* forming gel	An increased elimination of *Helicobacter pylori* compared to pexiganan alone. Minimal mucosal alterations and a lower amount of inflammatory cells in the stomach.	34
Delamanid	Tuberculosis	PT	Q_II_	Gel	556% bioavailability relative to the suspension.	35
		Selachyl alcohol	H_II_	Gel	645% bioavailability relative to the suspension.	

EE, encapsulation efficiency; GMO, glycerol monooleate; GDO, glycerol dioleate; H, hexagonal mesophase; PT, phytantriol; P407, poloxamer 407; PDI, polydispersity index; Q, cubic mesophase.

The LLC gels were adopted to improve oral bioavailability. Pexiganan is a natural peptide against *Helicobacter pylori* infections, albeit with poor solubility and poor metabolic stability. Incorporation in the hexagonal phase LLC gels increased the elimination effects of pexiganan to *Helicobacter pylori*^34^. The hexagonal phase protected pexiganan from harsh gastrointestinal conditions and prolonged its gastric retention owing to the bioadhesive effect. After 4 h of oral administration, only 26.5% of the administered pexiganan remained in the stomach from the suspension, whereas 72.80% remained from the LLC gels. Therefore, the pexiganan-loaded LLCs exhibited a superior curing effect on *Helicobacter pylori* infection in comparison to pexiganan alone.

The lipid type may affect the oral bioavailability of the LLC gels. PT and selachyl alcohol are non-digestible lipids, which form cubic and hexagonal phase gels of delamanid, respectively^35^. The cubic phase showed a 556% bioavailability relative to the delamanid suspension, whereas the hexagonal phase showed a 645% relative bioavailability. Administration with a digestible lipid (GMO) only achieved a relative bioavailability of 162% as the digestion of GMO did not improve the solubility of delamanid. However, PT- and selachyl alcohol-based systems were retained in the stomach for extended periods as the two lipids were resistant to digestive enzymes. As a result, the absorption of delamanid was prolonged, which significantly increased oral bioavailability. Therefore, the type of liquid crystal did not play a major role in the process.

## 3 Intravenous injection

Intravenous drug delivery is a cornerstone of modern medicine, offering a rapid onset of action, precise dosing, and the ability to bypass the limitations of absorption and metabolism associated with other delivery routes. However, conventional intravenous formulations often face challenges such as poor solubility, rapid clearance, systemic toxicity, and the lack of targeting specificity. LCNPs, particularly in the form of cubosomes and hexosomes, have emerged as a promising platform for overcoming these issues. Their unique mesophase structures allow encapsulation of both hydrophilic and hydrophobic drugs, ensuring efficient delivery and controlled release^36^. The recent advances focus on reducing off-target effects *via* surface modification and stimuli-responsive release (Table 2 37, 38, 39, 40, 41, 42).

**Table 2:** Advances of LCNPs in targeted drug delivery *via* intravenous injection.

Drug	Disease	Material	Phase	Properties	Achievement	Ref.
Copper acetylacetonate	LS174T colorectal cancer cells	GMO; P407; DSPE-PEG2000-azide; affimer protein	Im3m	Size: 106 nm; PDI: 0.18	A 5−7 fold higher drug distribution in the tumor tissue compared to the liver and kidney; A 5−7 fold higher accumulation in the tumor of the affimer-modified group compared to the nontargeted group.	37
Docosahexaenoic acid	Glioblastoma tumor	Phosphatidylglycerol; TPGS-mPEG2000	H_II_	Size: 90.4‒208.4 nm; Zeta potential: −12.4 to −58.5 mV	PEGylation prolonged the circulation time, lowered liver clearance, and increased accumulation in the brain.	38
Cisplatin; Temozolomide	Glioblastoma multiforme	GMO; DSPE-PEG3400-maleimide; Angiopep-2	Q_II_	Size: 261 nm; PDI: <0.3; Zeta potential:18.0 mV; EE: 73 ± 3% (temozolomide), 79 ± 1% (cisplatin)	A 3-fold increase in uptake by U87 cells and a 3-fold increase in accumulation within the brain than bare cubosomes without angiopep-2 modification.	39.
Formononetin	Colorectal cancer	GMO; P407; anisic acid	H_II_	Size: 127.0 ± 6.95 nm; PDI: 0.242 ± 0.015; Zeta potential: −24 ± 2.56; EE: 97 ± 2.8%	Higher cellular uptake than nanoparticles without anisic acid modification, leading to a higher apoptotic effect (33.55% *vs.* 22.88%), a higher tumor inhibition rate (79.00% *vs.* 58.75%), and a longer median survival (>30 days *vs.* 27 days).	40
Linoleoyl ethanolamide; oleoyl ethanolamide;	Arthritis	Synovia-targeted peptide HAP-1	Pn3m	Size: 170 nm; PDI: 0.124	Significantly reduced the pro-inflammatory cytokine levels in arthritic rats.	41
Panobinostat	Breast cancer	GMO; DSPE-PEG2000; P407; trastuzumab	Q_II_	Size: 185 nm; PDI: <0.2; Zeta potential: −37.6 ± 1.5 mV; EE: 34%	Higher cellular uptake and cytotoxicity were observed in HER2-positive SKBR3 cells compared with nanoparticles without trastuzumab modification and HER2-negative cells (L929 and 4T1).	42

DSPE-PEG2000, 1,2-distearoyl-*sn*-glycero-3-phosphoethanolamine-*N*-maleimide polyethylene glycol 2000; EE, encapsulation efficiency; GMO, glycerol monooleate; H, hexagonal mesophase; HER2, human epidermal growth factor receptor 2; P407, poloxamer 407; PDI, polydispersity index; PEGylation, polyethylene glycolylation; Q, cubic mesophase; TPGS-mPEG2000, d-*α*-tocopheryl succinate polyethylene glycol 2000.

### 3.1 Surface modification

Polyethylene glycolylation (PEGylation) and conjugation with ligands are involved in surface modification. PEGylation was generally achieved by incorporating d-*α*-tocopheryl succinate polyethylene glycol 2000 (TPGS-mPEG2000) or 1,2-distearoyl-*sn*-glycero-3-phosphoethanolamine-*N*-methoxy-polyethylene glycol 2000 (DSPE-mPEG2000) in the preparation process. The lipophilic chain was anchored to the lipid portion of the LCNPs, whereas the hydrophilic polyethylene glycol (PEG) was present on the surface to achieve long circulation capacity. With the help of activated PEG end, such as amino, maleimide, and azide, the ligands (*e.g.*, anisic acid, angiopep-2, affimer protein, and trastuzumab) were conjugated *via* click chemistry. When compared with the bare nanoparticles, the targeted nanoparticles exhibited a higher uptake by tumor cells and more distribution in the tumor tissues. The targeted nanoparticles, unexceptionally, enhanced tumor apoptotic effects and prolonged the median survival of tumor xenografted animals (Table 2). Intriguingly, the tumor tissues received a 5–7-fold higher distribution of affimer-tagged cubosomes relative to the liver and kidney^37^. This could be because of receptor-mediated endocytosis. It has been widely acknowledged that the high hepatic accumulation is a bottleneck to the applications of nanoparticles. The affimer-tagged cubosomes may provide an effective approach to changing the biodistribution of therapeutic nanoparticles.

In addition to monoolein, some active ingredients were adopted as constitutive structural components to develop LCNPs, enabling the unification of medicines and excipients. Docosahexaenoic acid has a broad therapeutic potential and forms hexosomes with phosphatidylglycerol (DOPG)^38^. The incorporation of either TPGS-mPEG2000 or DSPE-mPEG2000 realized the PEGylation of the nanoparticles, but a phase transition to a lamellar phase. PEGylation prolonged the circulation time, lowered liver clearance, and enhanced brain accumulation of the nanoparticles (Table 2). The *N*-acyl ethanolamines, such as linoleoyl ethanolamide (LEA) and oleoyl ethanolamide (OEA), are potent anti-inflammatory and analgesic agents. LEA and OEA could form stable LCNPs from cubic to lamellar phases, depending on the weight ratio^41^. Surface modification with a synovium-targeting peptide enhanced the joint distribution of the nanoparticles in both normal and arthritic rats, whereas LEA and OEA were liberated for the treatment of arthritics.

### 3.2 Stimuli-responsive LCNPs

**Figure 2:**
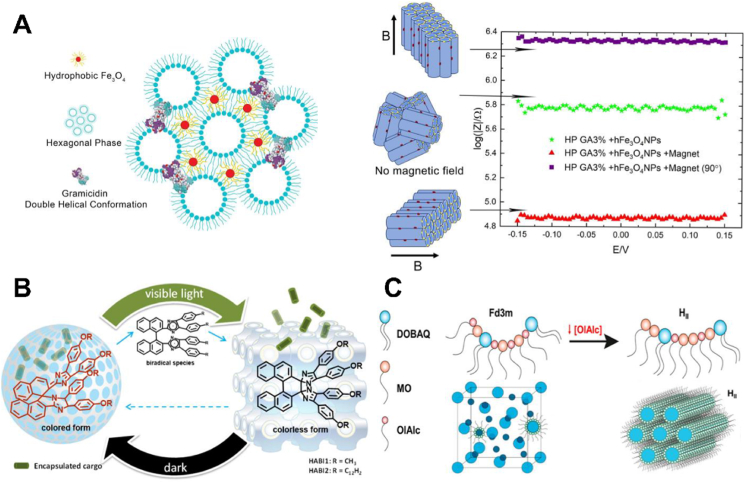
Stimuli-responsive LCNPs. (A) Fe_3_O_4_ superparamagnetic nanoparticles were incorporated into hexosomes to enable magnetically responsive. Reprinted with permission from Ref. 44. Copyright © 2024 Academic Press Inc Elsevier Science. (B) Light-induced phase transition of HABIs-functionalized LLC system for “on-demand” release. Reprinted with permission from Ref. 45. Copyright © 2020 Academic Press Inc Elsevier Science. (C) pH-induced phase transition by incorporating DOBAQ into cubosomes. Reprinted with permission from Ref. 46. Copyright © 2021 American Chemical Society.

Magnet, light, and pH are general stimuli used to enhance the targeting of specific disease sites. Fe_3_O_4_ superparamagnetic nanoparticles were incorporated into hexosomes to enable magnetically responsive^43^^,^^44^. Fe_3_O_4_ nanoparticles occupy the interstitial spaces within the hexagonal structure, leveraging the propensity of hydrophobic materials. Gramicidin adopts a binary helix configuration, thereby creating an ion channel that spans between two parallel lipidic channels. The incorporation did not alter the hexagonal mesophase. The magnetic field manipulates the alignment of the hexagonal structure. The alteration of the direction of the magnetic field by 90° induces a corresponding adjustment in the alignment of the hexagonal structure for on-demand drug release (Fig. 2A)^44^. The loading of hexaarylbiimidazoles (HABIs) endowed lamellar nanoparticles with light-controlled “on/off” release behavior^33^. HABI is a photochromic compound and undergoes an isomerization between the colored and the colorless form under visible light irradiation. The isomerization leads to a phase transition of HABIs loaded LLCs from a “slow-release” lamellar to a “fast-release” cubic phase, enabling a light-responsive release (Fig. 2B)^45^. HABIs revert to the initial conformation in the darkness that restores the system from the cubic to the lamellar phase (Fig. 2B)^45^. Incorporating pH-sensitive lipid *N*-(4-carboxybenzyl)-*N*,*N*-dimethyl-2,3-bis(oleoyloxy)propan-1-aminium (DOBAQ) into oleyl alcohol/monoolein cubosomes realized the pH-responsive release (Fig. 2C)^46^. DOBAQ becomes cationic at low pH (*e.g.*, ≤6), increasing its effective headgroup area and electrostatic repulsion between headgroups. The cubosomes are thus transformed into a porous hexagonal structure for the release of the cargo^46^.

## 4 Dermal drug delivery

Dermal drug delivery offers several advantages in treating systemic and localized diseases, including sustained release, ease of termination, painless application, and avoidance of first-pass metabolism. However, efficient transdermal delivery is challenging due to the barrier properties of the stratum corneum (SC). The SC is composed of 10–20 layers of dead corneocytes. A lipid matrix fills among the corneocytes to form a “brick-and-mortar” structure, preventing pathogens’ invasion as well as the entry of therapeutics, especially those with a molecular weight >500 Da^47^. Physical devices, penetration enhancers, and nanoparticles have been developed to overcome the SC barrier^48^^,^^49^. Among these approaches, LLCs have exhibited significant promise owing to their structural similarity to the SC lipids^20^. In addition, the unique properties, such as the strong encapsulating and sustained release capability, endow LLCs with a good platform for skin drug delivery^50^, ^51^, ^52^.

Despite the advantages of enhancing skin permeation, LLCs can promote wound healing by delivering anti-infective, angiogenic, and anti-inflammatory drugs. Wounds occur when the skin barrier gets damaged or compromised. Under normal conditions, the skin has an innate capacity for self-healing, progressing through the following four continuous phases: coagulation and hemostasis, inflammation, proliferation, and remodeling^53^. However, when the skin barrier is disrupted, the underlying tissue becomes susceptible to microbial contamination. Infectious wounds, particularly those associated with biofilms, pose significant treatment challenges owing to the increased resistance to conventional antibiotics. Biofilms, which are self-assembled clusters of microorganisms surrounded by an extracellular matrix, exhibit resistance to antibiotics that can be up to 1000 times greater than planktonic organisms^54^. Biofilm-related infections often lead to delayed or non-healing of wounds, necessitating innovative treatment strategies.

### 4.1 Enhanced skin permeation

Recent studies have highlighted the effectiveness of LLCs in treating local skin conditions, including atopic dermatitis, psoriasis, and melanoma (Table 3 55, 56, 57, 58, 59, 60, 61, 62, 63, 64, 65, 66, 67). Both LLC gels and LCNPs were involved. Among the different mesophases, the cubic phase was the most adopted. GMO was the most widely used material, but mainly led to the cubic phase LLCs. Monolinolein is flexible in preparing LLC mesophases by tuning its concentration in the ethanol aqueous solution^55^. A combination of oil (*e.g.*, hempseed/flaxseed oil, Olivem® 300, and oleic acid) and surfactants (*e.g.*, lecithin, tetradecyl glycoside sulfate, Procetyl™ AWS) was adopted to prepare different LLC mesophases^56^. Cubosomes are the main type of LCNPs (Table 3). Although the cubosomes were prepared from GMO and poloxamer 407 (P407), their sizes varied from 70 to 300 nm (Table 3). This variation may be attributed to the different preparation parameters.

**Table 3:** Advances of LLCs in dermal drug delivery.

Drug	Disease	Material	Phase	Properties	Achievement	Ref.
Gel						
TRP-2	Melanoma	Monolinolein (85%); 10% ethanol (15%)	L*α*	–	Ia3d has two times higher permeability than that of lamellar and Pn3d mesophases. A comparable vaccine effect to a subcutaneous injection without any skin irritation or cellular toxicity.	55
		Monolinolein (70%); 10% ethanol (30%)	Ia3d	–		
		Monolinolein (55%); 10% ethanol (45%)	Pn3d	–		
Nifedipine	–	Olivem® 300 (65%); Procetyl™ AWS (10%); water (25%)	L*α*	–	Flux: 0.678 ± 0.062 μg/cm^2^/h; lag: 1.4 h.	56
	–	Olivem® 300 (50%); Procetyl™ AWS (10%); water (40%)	L*α*/H_II_	–	Highest skin adhesion; flux 0.280 ± 0.038 μg/cm^2^/h; lag: 2.8 h.	
	–	Olivem® 300 (35%); Procetyl™ AWS (10%); water (45%)	H_II_	–	Highest skin permeation and retention. Permeation flux:1.022 ± 0.104 μg/cm^2^/h; lag: 3.2 h.	
Retinol	Skin inflammation	Sodium dodecyl; tetradecyl glycoside sulfate; oleic acid; polyethylene glycol 400	L*α*	–	Enhanced protection for retinol.	57
Octyl methoxycinnamate	Against UV radiation				Lower cutaneous permeability and higher retention in the stratum corneum.	
Betamethasone dipropionate	Atopic dermatitis	Lecithin; Tween 80; hempseed/flaxseed oil; water	L*α*	–	Repairing skin barrier; 2-fold reduction in transepidermal water loss and distinctive decrease in skin erythema levels.	58
Nanoparticle						
Apremilast	Psoriasis	GMO; Tween 20; P407	H_II_	Size: 180 ± 0.45 nm; EE: 89.42 ± 4.27%	7-Fold permeation flux and 9.4-fold skin retention than gel.	59
Dexamethasone acetate	Skin inflammation	GMO; P407	Q_II_	Size: 110.8 ± 0.87 nm; PDI: 0.167 ± 0.021; Zeta potential: −25.9 ± 1.31 mV	3.07 and 19.5 times increase in flux and intradermal retention than commercial preparation, respectively.	60
Apigenin	Melanoma	GMO; P407	Q_II_	Size: 287.7 ± 9.53 nm; PDI: 0.152 ± 0.051; EE: 80 ± 2.2%	2.4-Fold cumulative drug permeated, 2.8-fold AUC_0–24_ in epidermis, and 5-fold AUC_0–24_ in dermis than gel.	61
Apremilast	Psoriasis	GMO; Labrafil® M 2125 CS (linoleoyl Polyoxyl-6 glycerides); P407; water	H_II_	Size: 173.25 ± 2.192 nm; PDI: 0.273 ± 0.008; EE: 75.028 ± 0.235%	3.2- And 11.9-fold higher retention in stratum corneum and viable epidermis than gel, respectively. 8.4- and 2.06-fold higher AUC_0–24_ in the epidermis and dermis than gel, respectively.	62
Tenoxicam	Osteoarthritis	GMO; P407;	Q_II_	Size: <250 nm; PDI: <0.4; Zeta potential: −14.5 mV; EE: >90%	Flux: 23.1 μg/cm^2^/h; Percentage deposition after 6 h: 5.1 ± 1.5%; incorporating into hyaluronate gel didn’t affect flux but enhanced 5-fold skin deposition.	63
Fluocinolone acetonide	Atopic dermatitis	GMO; P407	Q_II_	Size: 265.3 nm; PDI: 0.428; Zeta potential: −47.3 mV; EE: 98.6%	Higher drug deposition (3.08 ± 0.16 μg/cm^2^) than the marketed formulation (1.1 ± 0.19 μg/cm^2^); a sustained release time of up to 24 h; improved efficacy and patient compliance.	64
Sulfasalazine	Rheumatoid arthritis	GMO; P407	Q_II_	Size: 65‒129 nm; Zeta potential: −18.8 to −24.8 mV; EE: 87%‒95%	3 times more deposition than the drug suspension.	65
Curcumin	Psoriasis	GMO; P407; polyethylene glycol 200	Q_II_	Size:126.03 ± 17.49 nm; PDI: 0.32 ± 0.01; EE: 88.25 ± 2.98%	2.57-Fold enhanced retention in stratum corneum as compared to gel.	66
Tofacitinib	Rheumatoid arthritis	GMO; myristol; P407	Q_II_	Size: 74.82 ± 4.26 nm; PDI: 0.124 ± 0.024; Zeta potential: −25 mV	The total amount permeated into the epidermis and dermis was 3.42 and 2.19 times greater than that of the cream, respectively	67

‒, not applicable. EE, encapsulation efficiency; GMO, glycerol monooleate. H, hexagonal mesophase; L*α*, lamellar mesophase; P407, poloxamer 407; PDI, polydispersity index; Q, cubic mesophase.

Both hydrophobic and hydrophilic drugs have been delivered to treat local diseases. Owing to the versatile loading capacity of LLCs, the encapsulating efficiencies all exceeded 60% irrespective of the hydrophilicity and hydrophobicity (Table 3). When compared with the commercial preparations or normal gels, LLCs significantly enhanced the permeation flux and skin retention in *in vitro* permeation studies. A maximum 7- and 19.5-fold increase in the permeation flux and skin retention was achieved in the delivery of apremilast and dexamethasone acetate by hexosomes and cubosomes, respectively^59^^,^^60^. In addition to the *in vitro* studies, dermatokinetics by measuring the drug concentration *vs.* time curves have been developed to precisely evaluate the skin retention *via* the area under the curve (AUC) value. For example, the apigenin-loaded cubosomes showed a 2.8- and a 5-fold higher AUC_0–24_ in the epidermis and dermis relative to a gel counterpart, respectively^61^; apremilast-loaded hexosomes showed an 8.4- and a 2.06-fold higher AUC_0–24_ in the epidermis and dermis compared to a gel counterpart, respectively^62^. The increased skin retention benefits the treatment of local lesions.

Monolinolein-based LLCs enhanced the skin permeation of a peptide antigen, TRP-2, to obtain a better therapy for melanoma^55^. Monolinolein forms lamellar, Ia3d, and Pn3m phases, depending on the concentration. All LLCs enhanced the skin permeability of fluorescein isothiocyanate (FITC)-labeled TRP-2. Conversely, for the aqueous solution and the hydroxyethyl cellulose gel, the fluorescent signal remained at the SC layer and could not be observed in the deeper skin. Intriguingly, Ia3d mesophase exhibited 2-fold higher permeability than that of the lamellar and Pn3m geometries. The Ia3d mesophase even showed a comparable vaccine effect to a subcutaneous injection. It was estimated that the Ia3d mesophase could deliver the antigen to dendritic cells in the epidermal layer, thereby stimulating a stronger immune response and a significant reduction in the growth of melanoma.

In addition to the therapeutic application, lamellar LLCs could impregnate octyl methoxycinnamate in the skin for sunscreen^57^. The viscoelasticity of the lamellar mesophase contributed to the permanence at the site of use. The resemblance to SC lipids retained octyl methoxycinnamate in SC after the entrance. The controlled-release capacity of LLCs ensured a minimal systemic delivery. In addition, the LLCs enhanced the stability of the co-loaded retinol^57^. During the 4-week storage period at 32 °C, 71.16% retinol remained in the gel, surpassing the protection capacity of nanoparticles. The lamellar phase provides greater protection than scattered structures, considering that they are more effective in cutting off from the environmental degrading factors (such as O_2_ and heat).

Lamellar LLCs *per se* could repair impaired skin barrier, thereby assisting in the treatment of inflammatory skin diseases^58^. The intercellular lipids form the SC barrier, which is organized into lamellar phase structures. The distinctive structural organization prevents xenobiotic penetration and excessive transepidermal water loss (TEWL). Impaired integrity of the SC represents a precondition and a common feature in inflammatory skin diseases^68^. The lamellar mesophase is extremely beneficial in repairing skin barrier dysfunction owing to its resemblance to SC lipids. Hempseed/flaxseed oil is abundant in essential fatty acids that benefit the complementation of deficient lipid composition in inflammatory skin. Therefore, hempseed/flaxseed oil-based lamellar mesophase reduced TEWL by 2-fold and restored the hydration of the skin surface^58^. The recovered homeostasis is positive in reducing skin erythema.

Topical preparations should be viscoelastic for ease of application. Therefore, the LCNPs were incorporated into hyaluronate gels^63^. However, there needs to be a balance between viscoelasticity and skin permeability. As high viscoelasticity creates a formidable barrier within the matrix, it often signifies a significant risk of impaired drug diffusion and reduced skin permeability. However, there is no universal threshold for viscoelasticity, as it varies depending on the mesophase of the LLCs and the properties of the loaded drugs. The formulation should be optimized to achieve the minimum viscoelasticity necessary for stability and controlled release while maximizing permeation, as guided by rheological characterization coupled with permeation studies. Intriguingly, hyaluronate gels did not affect the permeation flux of tenoxicam-loaded cubosomes; instead, the gel showed a 5-fold increase in the skin deposition of tenoxicam relative to cubosomes alone^63^. The underlying reasons remain unraveled.

### 4.2 The mechanisms for enhanced skin permeation

The ability of LLCs to enhance skin permeability can be attributed to their amphiphilic molecules, such as GMO, monolinolein, fatty acids-enriched oils, and surfactants like Tween and poloxamer. They are the permeation enhancers that attenuate the SC layer and/or disrupt the SC lipids. Histopathological studies have demonstrated that LLCs reduce the thickness of the SC and weaken the intracellular junctions between corneocytes^69^. Differential scanning calorimetry (DSC) and Fourier-transformed infrared spectroscopy (FTIR) confirmed the disrupting effects of LLCs on the SC^70^. The normal SC exhibited an endothermic peak at 174.998 °C, which can be attributed to protein denaturation. Conversely, SC treated with LLCs displayed a pronounced endothermic peak at 115.073 °C. This lower peak indicates a loss of the SC juncture, which is essential for enhancing drug penetration^70^. The normal skin presented FTIR bands at 2921.41 and 2854.49 cm^−1^, which can be attributed to the asymmetric and symmetric C–H stretching in the lipids, respectively. FTIR bands at 1645.36 and 1536.74 cm^−1^ may be attributed to the proteins’ amide I and amide II stretching vibrations, respectively. The spectra of the treated skin received a slight shift from that of the normal skin, that is, 2858.82 cm^−1^ of the C–H stretching, 1723.62 cm^−1^ of the amide I stretching, and 1546.83 cm^−1^ of the amide II stretching, respectively^70^. These peak variations confirmed that LLCs weakened the barrier function *via* their interactions with the SC lipids.

The mesophase of LLCs plays a crucial role in their interaction with the SC. Past studies using FTIR have demonstrated that LLCs with Ia3d geometry induced greater blue shifts for symmetric and asymmetric vibration of C–H of the SC sheet than the lamellar and the Pn3m mesophases^55^. The blue shifts indicated that the structural disruption of the SC lipid reduced the orientational order. Conversely, treatment with 10% ethanol and cellulose gel exhibited no changes. These findings were highly consistent with the skin permeation.

The disruption effect is more pronounced with amphiphilic molecules with higher degrees of unsaturation. Tween 80 containing LLCs displayed a stronger intensity compared to those containing Poloxamer 127^58^. The enhancers extracted the intercellular lipids in the SC and disrupted the keratin filaments. Enhancers with higher unsaturation degrees disrupt the skin barrier more strongly. Monolinolein presents stronger enhancing effects compared to monoolein and monostearin. Therefore, monolinolein-based LLCs showed higher amounts of TRP-2 in murine skin than monoolein-based LLCs^55^.

**Figure 3:**
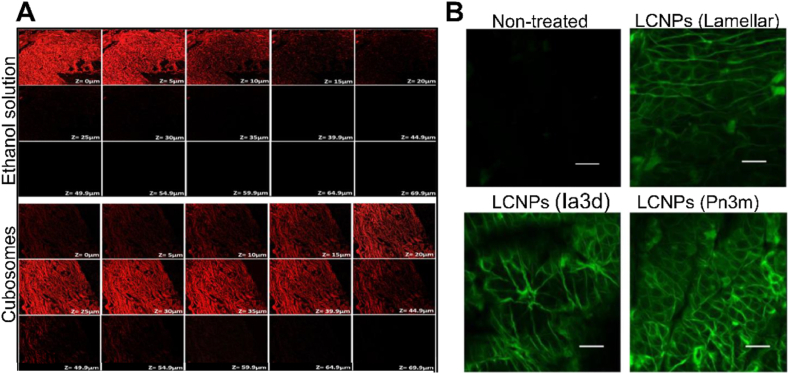
Confocal laser scanning microscopy images of mouse skin treated with different formulations. (A) Rhodamine-loaded cubosomes permeate up to 59.9 μm in the skin, while the rhodamine solution only reaches 20 μm (red signal indicating rhodamine). Reprinted with permission from Ref. 61. Copyright © 2022 Elsevier. (B) FITC-labeled LCNPs localize in the intercellular spaces of the keratinocytes (green signal indicating FITC), scale bar = 50 μm. Reprinted with permission from Ref. 55. Copyright © 2022 Elsevier.

Confocal laser scanning microscopy (CLSM) revealed the depth and route of LCNPs penetrating the skin (Fig. 3A)^61^. Rhodamine-loaded cubosomes permeate up to 59.9 μm in the skin, whereas the rhodamine solution only reached 20 μm^61^. In addition, the *Z*-axis scan of the skin treated by monolinolein-based LLCs displayed fluorescent signals in the intercellular spaces of the keratinocytes, indicating the paracellular route of permeation (Fig. 3B)^55^.

In summary, LLCs enhance skin permeation primarily by fluidizing the SC lipid matrix and extracting intercellular lipids, thereby compromising the SC barrier function. The efficacy of this process is governed by two critical factors: the chemical properties of amphiphilic components (*e.g.*, degree of unsaturation) and the internal mesophase geometry. Amphiphiles with higher unsaturation (*e.g.*, monolinolein > monoolein) synergize with optimal mesophases (*e.g.*, cubic Ia3d geometry) to maximize permeation enhancement. In addition, particle size modulates the penetration depth by favoring paracellular routes, as demonstrated by confocal imaging of intracellular LCNP trafficking. Smaller LCNPs formulated with optimal amphiphiles and mesophases demonstrate maximum permeation enhancement owing to synergistic barrier disruption and efficient paracellular trafficking.

### 4.3 Wound healing

LCNPs have demonstrated promising results in enhancing the antibacterial and antibiofilm activities of positively charged antibiotics (Table 4 71, 72, 73, 74, 75, 76, 77, 78, 79, 80, 81). For instance, cationic aminoglycosides, which are commonly used for antipseudomonal therapy, have limited effectiveness against *Pseudomonas aeruginosa* biofilms as they are bound to the biofilm matrix’s negatively charged polysaccharides, thereby preventing penetration into the inner bacterial community. However, the incorporation of tobramycin into monoolein-based LCNPs has exhibited a 1000-fold increase in antimicrobial activity against *Pseudomonas aeruginosa* biofilms when compared to an antibiotic solution^72^. The unique liquid crystal structure, nano-sized particles, and negative surface charge of LCNPs enhance the penetration of the antibiotic through a biofilm barrier. In addition, these LCNPs exhibit a strong fusion efficiency with planktonic *Pseudomonas aeruginosa*, which further increases the cellular uptake of the antibiotic. Similarly, monoolein-based LCNPs reduced the minimum inhibitory concentrations of amikacin and gentamicin to *Pseudomonas aeruginosa* by 2-fold and increased the antimicrobial activity of both antibiotics against *Pseudomonas aeruginosa* biofilms by 100-fold when compared to the solution^73^. Co-loading with glycoside hydrolase (PSlG) further enhanced the antimicrobial effect of tobramycin by 10‒100 folds^74^. The liquid crystal structure protects PSlG from protease degradation. However, the bacterial lipases triggered the release of the enzymes from the monoolein-based LCNPs, which selectively degraded the polysaccharide in *Pseudomonas aeruginosa* biofilms. Then, the biofilm was dispersed into planktonic bacteria, thereby reviving the susceptibility to tobramycin.

**Table 4:** Advances of LLCs in antiinfection and wound healing.

Drug	Disease	Material	Phase	Properties	Achievement	Ref.
Simvastatin	Wound healing	GMO; P407; Hydroxypropyl methylcellulose	Q_II_	Size: 80‒183 nm; PDI: 0.14‒0.39; Zeta potential: −8.2 to −26.1 mV; EE: 60.5%‒93.95%; loading: 2.25%‒10.20%	1.2‒1.8 fold increase in the cumulative amount permeated, transdermal rate, and the apparent permeability coefficient than free drugs. Higher wound closure percentage than free drugs after 11 days (92.32 ± 1.61% *vs.* 77.00 ± 2.26%).	71
Tobramycin	*Pseudomonas aeruginosa* biofilm	GMO; P407	–	Size: 164 ± 2 nm; PDI: 0.16 ± 0.01; Zeta potential: −10.4 ± 0.04 mV; EE: 85%; loading: 25%	A 1000-fold enhancement in antimicrobial activity compared to a solution due to enhanced penetration across the biofilm barrier and fuse with *Pseudomonas aeruginosa* cells.	72
Amikacin	*Pseudomonas aeruginosa* infection	GMO; P407	–	Size: 170 ± 4 nm; PDI: 0.08 ± 0.01; Zeta potential: −9.76 ± 0.8 mV; loading: 25%	Reduce MIC from 2 μg/mL of free drug to 1 μg/mL. A 100-fold increase in the antimicrobial activity against *Pseudomonas aeruginosa* biofilms compared to the solution.	73
Gentamicin				Size: 167 ± 9 nm; PDI: 0.18 ± 0.02; Zeta potential: −10.6 ± 0.3 mV; loading: 25%	Reduce MIC from 1 μg/mL of free drug to 0.5 μg/mL. A 100-fold increase in the antimicrobial activity against *Pseudomonas aeruginosa* biofilms compared to the solution.	
PSlG; tobramycin	*Pseudomonas aeruginosa* biofilm	GMO; P407	–	Size: 182 ± 3 nm; PDI: 0.38 ± 0.02; Zeta potential: −14 ± 0.4; EE: 93.9%; loading: 1.5% (PSlG), 5.6% (tobramycin)	Protect PSlG from proteolysis; triggered and sustained release in the presence of bacteria; 10- to 100-fold improvement in the antimicrobial effect of tobramycin.	74
Gallium protoporphyrin	*Pseudomonas aeruginosa* biofilm	GMO; P407	Q_II_	Size: 156 ± 1.4 nm; PDI: 0.21 ± 0.02; Zeta potential: −29.9 ± 0.91 mV	2-Fold enhancement in the performance of photosensitizer; 500 times increase in antimicrobial activity against *Pseudomonas aeruginosa* biofilm compared to unformulated compound.	75
Gallium protoporphyrin	*Staphylococcus aureus*-infection	GMO; P407	–	Size: 178 ± 3.1 nm; PDI: 0.19 ± 0.04; Zeta potential: −30.5 ± 2.2 mV; EE: 98.3 ± 4.3%; loading: 3.3 ± 0.3%	A 700 times increase in the *in vitro* antimicrobial activity against *Staphylococcus aureus* biofilms and 72% improvement in photodynamic activity compared to the unformulated compound.	76
Docosahexaenoic acid	*Staphylococcus aureus* biofilm	Phosphatidylglycerol	H_II_	Size: 180.10 ± 2.1 nm; PDI: 0.29 ± 0.10; Zeta potential: −62.8 ± 2.4 mV	A five-fold reduction of the planktonic and a four-fold reduction of biofilm populations of *Staphylococcus aureus*.	77
N-terminal palmitoylated Arg-rich tetrapeptide	Focal bacterial infections	GMO; ethanol	Q_II_	*In situ* forming gel	Better *in vivo* antibacterial activity and long-term inhibition effects than free drugs. A 99.4% reduction in bacterial load in infected mouse skin after five days of treatment.	78
*ε*-polylysine	Infected post-operative wounds	GMO; ethanol; polyethylene glycol 400	L*α* to Q_II_	*In situ* forming gel	More than 99% methicillin-resistant *Staphylococcus aureus* were killed in 3 h. The antibacterial effect lasted for 48 h. Less than 0.1% bacteria survived on Day 7 with wound closure higher than 90%.	79
LL37; carbenoxolone	Diabetic wound	GMO; polyethylene glycol 400	Q_II_	*In situ* forming gel	The rapid release of LL37 achieved rapid wound sterilization. Carbenoxolone assisted LL37 in reducing the inflammation level of the wound from the inside.	80
*β*-sitosterol	Thermal burn	GMO; oleic acid; P407 Tween 80; Hydroxypropyl methylcellulose	Q_II_	Size: 74.4 ± 2.8 nm; PDI: 0.367 ± 0.02; Zeta potential: −29.6 ± 1.1 mV; EE: 92.3 ± 3.8%	Increasing its skin penetration of *β*-sitosterol and keeping the burn moisture. Significantly higher wound closure percentage relative to Mebo® on the Day 14, ∼90% *vs.* ∼70%.	81

‒, not applicable. EE, encapsulation efficiency; GMO, glycerol monooleate; H, hexagonal mesophase; L*α*, lamellar mesophase; LL37, human antimicrobial peptides; MIC, minimum inhibitory concentration; P407, poloxamer 407; PDI, polydispersity index; PSlG, glycoside hydrolase; Q, cubic mesophase.

LCNPs can improve the antibacterial activity of lipophilic antibiotics. Gallium protoporphyrin, a photosensitizer that generates reactive oxygen species upon activation, has exhibited enhanced activity against bacterial biofilms when delivered by monoolein-based LCNPs^75^^,^^76^. These nanoparticles increased the antimicrobial activity of gallium protoporphyrin against *Pseudomonas aeruginosa* biofilms by 500-fold and against *Staphylococcus aureus* biofilms by 700-fold when compared to the unformulated compound. This enhanced activity can be attributed to the nanoparticles' ability to overcome the biofilm matrix and deliver the photosensitizer more effectively to bacterial cells. Furthermore, LCNPs can enhance the effectiveness of docosahexaenoic acid (DHA), which is a nutraceutical showcasing broad-spectrum antimicrobial activities by altering the structure and composition of bacterial cell membranes. Lipophilicity hinders DHA’s access to microbes within the biofilm matrix. Hexosomes could deliver DHA across the biofilm of *Staphylococcus aureus* and achieved a four-fold increase in biofilm eradication^77^.

LLC gels have been explored for wound healing because they form a moist environment conducive to tissue regeneration, sustained drug release, and a biomimetic structure for cell growth. The liquid crystal-forming materials can be dissolved in ethanol or polyethylene glycol as a precursor. Following spraying in the wound area and the absorption of the solution, the LLC gels formed *in situ*. Antimicrobial peptides, such as N-terminal palmitoylated Arg-rich tetrapeptide (C16R4), *ε*-polylysine, and human antimicrobial peptides (LL37), were delivered by the *in situ* forming gel for wound healing^78^, ^79^, ^80^. These gels provide rapid wound sterilization, killing over 99% of the bacterial load within hours, while maintaining antibacterial activity over several days. Wound closure was >90% on Day 7, consequently^79^. Meanwhile, carbenoxolone was co-loaded to reduce the inflammation level of the wound from the inside, thereby further promoting wound healing^80^.

When incorporated into hydrogels, cubosomes achieved a prolonged residence time covering the wound site and demonstrated superior performance relative to unformulated drugs. For example, the cubosome-based hydrogels significantly accelerated the wound-healing effects of hesperidin, simvastatin, and *β*-sitosterol^70^^,^^71^^,^^81^. These drugs benefit wound recovery due to the angiogenic and anti-inflammation effects, albeit their effects are limited by poor solubility in water. The cubosome-based hydrogels not only enhanced their solubility but also enhanced their permeability. The transdermal rate of simvastatin was increased by 1.2‒1.8 fold, depending on the composition of the cubosomes^71^.

## 5 Ocular drug delivery

Effective ocular drug delivery is challenged by multiple anatomical and physiological barriers, including tear dilution, nasolacrimal drainage, blinking, and the corneal epithelium. The cornea comprises three distinct layers, a lipophilic epithelium, a hydrophilic stroma, and a lipophilic endothelium, forming a “sandwich” structure that limits the penetration of both hydrophilic and lipophilic drugs. As a result, conventional eye drops achieve less than 5% bioavailability^82^. Enhancing drug retention on the ocular surface and facilitating transcorneal permeation is therefore critical to improving therapeutic efficacy^83^.

Cubosomes have shown significant promise in overcoming these barriers (Table 5 84, 85, 86, 87, 88, 89, 90, 91). Typically prepared using GMO or PT as the lipid matrix and stabilized by surfactants such as Poloxamer or polyvinyl alcohol, cubosomes offer high structural versatility. The incorporation of **limonene**, a known permeation enhancer, further improves corneal penetration by transiently disrupting the lipid bilayer of the corneal epithelium, reducing membrane rigidity and increasing permeability^84^^,^^92^. Due to their versatile loading capacity, cubosomes have been successfully used for delivering poorly soluble antibiotics, such as ciprofloxacin, voriconazole, and vancomycin, for treating ocular infections like keratitis, as well as hydrophilic antiglaucoma drugs for treating glaucoma (Table 5).

**Table 5:** Advances of LLCs in ocular drug delivery.

Drug	Disease	Material	Phase	Properties	Achievement	Ref.
Fenticonazole nitrate	Ocular fungal infections	GMO; poloxamer 188; limonene; polyvinyl alcohol	Q_II_	Size: 249.75 ± 1.48 nm; PDI: 0.38 ± 0.09; Zeta potential: −34.25 ± 0.64 mV; EE: 87.94 ± 0.7%	A 2.69-fold enhancement in the transcorneal permeation than suspension.	84
Luteolin	Glaucoma	GMO; P407; chitosan	Q_II_	Size: 258 ± 9.05 nm; PDI: 0.26 ± 0.04; Zeta potential: 49 ± 6.09 mV; EE: 95 ± 2.14%	A 3.60-fold higher transcorneal permeation than suspensions. 6.46- and 1.89-fold reductions in intraocular pressure compared to suspensions and uncoated nanoparticles.	85
Ciprofloxacin	Conjunctivitis and corneal ulcers	PT; P407; chitosan	Q_II_	Size: 121 nm; EE: 75%	A 2.54-fold increase in antimicrobial activity than eye drops. Once-daily administration maintained the effective drug concentration in the aqueous humor.	86
Voriconazole	Ocular fungal infections	GMO; P407; chitosan	Q_II_	Size: 243 ± 12.29 nm; PDI: 0.25 ± 0.01; Zeta potential: −32.78 ± 3.85 mV; EE: 90.58 ± 0.48%	Chitosan coating provided high mucoadhesive properties. 1.75-time extension in the period during which the aqueous humor concentrations were at least 50% of the peak concentration. A relative bioavailability of 171%.	87
Natamycin	Ocular fungal infections	PT; Acrylates/C10-30 alkyl acrylate crosspolymer; Carbopol 934; Hydroxypropyl cellulose	Q_II_	Size: 57 nm; PDI:0.3; Zeta potential: 22 mV; EE: 82%	A 5.2-fold enhancement in the cumulative amount permeated and flux than suspension.	88
Vancomycin	Bacterial keratitis	GMO; P407	Q_II_	Size: 51.11 ± 0.96 nm; PDI: 0.275 ± 0.53; Zeta potential: −25.94 ± 0.68 mV; EE: 91.624 ± 0.42%	A 2.4-fold increase in apparent permeability, 1.9-fold higher *T*_max_, 2.2-fold greater mean residence time, and 2.23-fold higher AUC than solution.	89
Ketoconazole	Ocular fungal infections	GMO; P407; polyvinyl alcohol; Carbopol 971P	Q_II_	Size: 268.9 ± 14.58 nm; PDI: 0.437 ± 0.032; EE: 38.63 ± 1.32%; loading: 0.2%	A 5-time higher drug permeation and 6-fold higher skin deposition than the marketed cream.	90
Acetazolamide	Glaucoma	GMO; P407; polyvinyl alcohol	Q_II_	Size: 243 ± 4.2 nm; PDI: 0.23 ± 0.03; Zeta potential: −26.1 ± 0.6 mV; EE: 73.99%	A 2.02-fold enhancement in the cumulative percentage of drug permeation through the goat cornea and a 2.96-fold increase in corneal concentration than the solution.	91

AUC, area under the curve; EE, encapsulation efficiency; GMO, glycerol monooleate; PT, phytantriol; P407, poloxamer 407; PDI, polydispersity index; Q, cubic mesophase; *T*_max_, time to peak drug concentration.

**Figure 4:**
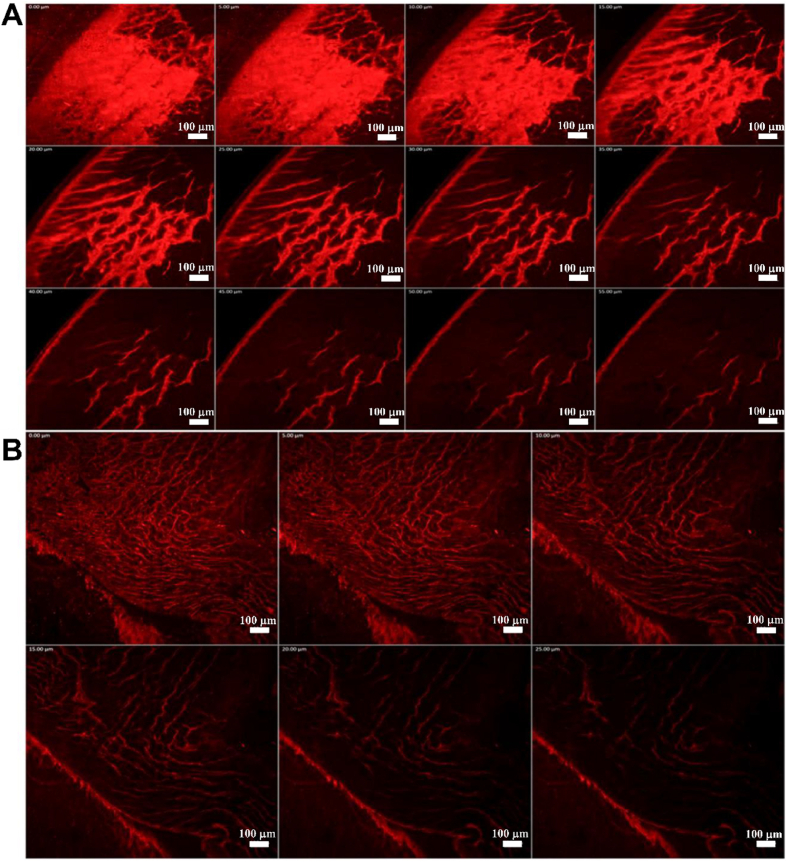
Confocal laser scanning microscopy image of the cornea of rabbits treated by (A) rhodamine-loaded cubosomes and (B) rhodamine aqueous solution, scale bar = 100 μm. Reprinted with permission from Ref. 84. Copyright © 2024 Elsevier.

Their enhanced permeability is attributed to two main factors: the **cubic mesophase** and **nanoscale particle size**. The internal cubic structure mimics the lipidic organization of epithelial membranes, promoting membrane fusion and drug transport^11^, while the small particle size facilitates traversal through the layered architecture of the cornea^93^. Additionally, the **mucoadhesive properties** of cubosomes prolong ocular residence time, allowing for greater absorption and therapeutic effectiveness^94^. The successful transcorneal transport of intact cubosomes was confirmed *via* CLSM, using rhodamine B as a fluorescent probe^84^. Rhodamine B-loaded cubosomes exhibited deeper penetration (55 μm) compared to a simple solution (25 μm), further demonstrating their enhanced delivery potential (Fig. 4)^84^.

Chitosan coating was adopted to prolong the corneal residence time of cubosomes^85^, ^86^, ^87^. As a cationic polysaccharide, chitosan reverses the surface charge of cubosomes from negative to positive (Table 5), enabling electrostatic interactions with the negatively charged mucin layer of the tear film. This interaction significantly improves mucoadhesion and ocular retention. For instance, chitosan-coated cubosomes extended the antiglaucoma activity of luteolin beyond 24 h, whereas uncoated formulations sustained the effect for only ∼5 h^85^. Similarly, once-daily administration of chitosan-coated ciprofloxacin-loaded cubosomes maintained therapeutic drug levels in the aqueous humor, whereas the commercial formulation required four daily doses to achieve comparable concentrations^86^. In another study, chitosan coating prolonged the duration during which aqueous humor concentrations of voriconazole remained above 50% of the peak by 1.75 times compared to uncoated cubosomes^87^.

Incorporating hydrogels is another strategy to increase ocular residence. Carbopol is a widely used gel matrix due to its strong mucoadhesive properties and favorable rheological behavior^95^. For example, Carbopol 934 was used as a pH-responsive polymer in the development of an *in situ* gel containing natamycin-loaded cubosomes^88^. Upon exposure to tear fluid, the formulation undergoes a sol-to-gel transition, enhancing drug retention. Hydroxypropyl cellulose was additionally incorporated to further increase mucoadhesion, thereby supporting prolonged residence time and sustained drug release.

## 6 Intranasal administration

Intranasal drug delivery represents a promising strategy for treating neurological disorders by enabling direct drug transport from the nasal cavity to the brain *via* the olfactory and trigeminal pathways^96^. Neurological disorders are the second leading cause of global disability and mortality and are projected to rise substantially by 2050^97^. Despite the advantage of bypassing the blood–brain barrier, the effectiveness of intranasal delivery is often limited by rapid mucociliary clearance, which reduces drug residence time in the nasal cavity^98^. The bioadhesive nature of LLCs, along with their capacity for versatile drug loading, controlled release, and permeability enhancement, makes them particularly suitable for overcoming these barriers in nasal-to-brain delivery^99^. The advances in intranasal administration of LLCs are summarized in Table 6 100, 101, 102, 103, 104, 105, 106.

**Table 6:** Advances of LLCs in intranasal administration.

Drug	Disease	Material	Phase	Properties	Achievement	Ref.
Tizanidine hydrochloride	Muscular spasm	GMO; P407; ethanol; polyethylene glycol 200;	Q_II_	Size: 50.22 nm; Zeta potential: −6.39 mV; EE: 69.28%	Pharmacokinetics in plasma: 2.59-fold lower *C*_max_ and 1.21-fold lower AUC than the tablet. Pharmacokinetics in the brain: 2.15-fold higher *C*_max_ and 1.51-fold higher AUC than the tablet.	100
Plasmalogen	Parkinson’s disease	1,2-Dioleoyl-3-trimethylammonium propane; vitamin E; P407	H_II_	–	Improve the behavioral Parkinson’s disease symptoms and downregulate IL-6, IL-33, and TNF-*α* genes.	101
Fluoxetine	Depression	GMO; glycerol tripalmitate; P407; hydroxypropyl methylcellulose	Q_II_	Size: 149.8 nm; PDI: 0.118; Zeta potential: 16.23 mV	Sustained release lasts for 24 h. A 2.21- and 2.83-fold increase in brain distribution than cubosomal dispersion and drug solution, respectively.	102
Verapamil hydrochloride	Cluster headache	GMO; P407;	–	Size: 210.49 nm; Zeta potential: −30.34 mV; EE: 77.24%	Sustained release lasts for 24 h. A 2.26-fold increase in the flux and the permeability coefficient than the solution. Pharmacokinetics in plasma: 1.37-fold higher *C*_max_ and 1.58-fold higher AUC than the solution (intranasal); 1.42-fold lower *C*_max_ and 1.07-fold higher AUC than the solution (intravenous). Pharmacokinetics in the brain: 1.30-fold higher *C*_max_ and 1.98-fold higher AUC than the solution (intranasal); 1.62-fold higher *C*_max_ and 2.61-fold higher AUC than the solution (intravenous).	103
Donepezil	Alzheimer’s disease	Polyethylene glycol hexadecyl ether; oleic acid; water	H_II_	–	The phase transition from a water/oil microemulsion to H_II_ mesophase, adhering strongly to the nasal mucosa, increasing the residence time, and sustaining donepezil release. A 23.93-fold higher AUC in the brain than in plasma; A 16.67-fold higher *C*_max_ in the brain than in plasma.	104
Vinpocetine	Vascular dementia	GMO; oleic acid; P407	H_II_	Size: 102.95 ± 1.85 nm; PDI: 0.531 ± 0.01; Zeta potential: −30.4 ± 0.1 mV; EE: 87.40 ± 1.37%	Pharmacokinetics in plasma: 16.09-fold higher *C*_max_ and 3.39-fold higher AUC than the intravenously injected solution; Pharmacokinetics in the brain: 12.67-fold higher *C*_max_ and 12.57-fold higher AUC than the intravenously injected solution; A 4.99-fold higher AUC in the brain than in plasma.	105
		GMO; oleic acid; P407; gellan gum			Pharmacokinetics in plasma: 1.63-fold lower *C*_max_ and 8.86-fold higher AUC than the intravenously injected solution; Pharmacokinetics in the brain: 1.04-fold lower *C*_max_ and 4.62-fold higher AUC than the intravenously injected solution; A 6.47-fold higher AUC in the brain than in plasma.	
Repaglinide	Diabete	GMO; Myrj 59	Q_II_	Size: 170 ± 4.54 nm; PDI: 0.343 ± 0.041; Zeta potential: −4.90 ± 0.51 mV; EE: 93.89 ± 1.86%	Maximum reduction in blood glucose level: 66.35 ± 2.07%; pharmacological availability: 178.26 ± 2.61%	106
		GMO; Myrj 59; P407; poloxamer 188			Maximum reduction in blood glucose level: 38.88 ± 2.78%; pharmacological availability: 180.96 ± 4.34%	

‒, not applicable. AUC, area under the curve; *C*_max_, peak plasma concentrations; EE, encapsulation efficiency; GMO, glycerol monooleate; H, hexagonal; P407, poloxamer 407; PDI, polydispersity index; Q, cubic.

Recent research has focused on the use of LCNPs for enhancing intranasal drug delivery to treat neurological disorders, *e.g.*, muscular spasms^100^, Parkinson’s disease^101^, depression^102^, cluster headaches^103^, Alzheimer’s disease^104^, and vascular dementia^105^. Among them, GMO-based cubosomes are the main carrier for intranasal delivery^100^^,^^102^^,^^103^. Notably, all cubosomes achieved a sustained release lasting for 24 h. Cubosomes could enhance the permeability of co-formulated drugs. For instance, the permeability of verapamil hydrochloride across sheep nasal membranes was increased by 2.26-fold when being loaded in cubosomes^103^. Consequently, cubosomes yielded higher peak plasma concentrations (*C*_max_) and AUC for verapamil in both plasma and brain tissue relative to intranasal solution. Notably, brain accumulation of verapamil was also higher with intranasal cubosomes than with intravenous administration, despite lower plasma exposure. Similar trends were observed for tizanidine and fluoxetine, where intranasal cubosomes enhanced brain targeting while reducing systemic exposure compared to oral administration^100^^,^^102^. These findings underscore the potential of cubosomes as effective carriers for intranasal brain-targeted drug delivery.

Combining LCNPs with hydrogels has been shown to further enhance nose-to-brain drug delivery. For instance, GMO-based hexosomes alone resulted in a 4.99-fold higher AUC of vinpocetine in the brain compared to plasma following intranasal administration^105^. However, this formulation also led to a 16.09-fold higher *C*_max_ and a 3.39-fold higher AUC in plasma than the intravenously administered solution, raising potential toxicity concerns. When hexosomes were incorporated into a gellan gum hydrogel, both plasma and brain *C*_max_ values were reduced relative to intravenous injection, while brain AUC increased by 4.62-fold. Notably, this gel-based formulation achieved a 6.47-fold higher brain AUC than plasma AUC, indicating improved brain targeting with reduced systemic exposure.

An *in situ* forming LLC gel was developed to increase nose-to-brain delivery of donepezil to treat Alzheimer’s disease^104^. This system was formulated as an oil-in-water microemulsion comprising polyethylene glycol hexadecyl ether, oleic acid, and water. Upon contact with nasal fluid, the formulation underwent a phase transition into the H_II_ mesophase, thereby increasing mucosal residence time. As a result, the gel achieved a 23.93-fold higher AUC and a 16.67-fold higher *C*_max_ in the brain than in plasma.

Besides neurological disorders, cubosomes have also been explored for intranasal delivery of repaglinide, a short-acting anti-hyperglycemic agent with significant first-pass metabolism^106^. Compared to intravenous injection, which caused a rapid hypoglycemic effect within 0.5 h but returned to baseline glucose levels by 4 h, the intranasal cubosomes exhibited a sustained hypoglycemic response from 2 to 24 h. This formulation yielded a pharmacological availability of 178.26 ± 2.61%. The incorporation of the cubosomes into a thermoresponsive *in situ* gel composed of P407 and P188 further modulated the release profile. Although this gel formulation reduced the maximum decrease in blood glucose levels from 66.35 ± 2.07% to 38.88 ± 2.78%, it maintained a high pharmacological availability (180.96 ± 4.34%), indicating a more stable and prolonged therapeutic effect.

## 7 Local implantation for long-acting therapy

The long-term management of chronic diseases necessitates the development of drug-delivery systems capable of sustained release and improved pharmacokinetic profiles. Local implantation has emerged as a promising strategy for long-acting drug delivery, as it can prolong therapeutic effects and enhance patient adherence^107^. These systems offer several advantages, including reduced dosing frequency and controlled, sustained drug release, thereby improving treatment efficacy^108^. Current clinical applications include biodegradable polymer-based microparticles, multivesicular liposomes, micro- and nanocrystal suspensions, and oil-based formulations^109^^,^^110^. Despite their potential, these delivery systems also present certain limitations. Suspension and oil-based formulations are typically limited to a narrow range of poorly soluble drugs and face challenges in modulating drug release kinetics^111^^,^^112^. Polymeric systems currently represent the predominant long-acting formulation strategy, accounting for approximately 62% of the market, and rely on matrix degradation to control drug release^112^. However, their broader clinical application is hindered by issues such as complex manufacturing processes, risk of needle blockage, and the need for surgical implantation.

### 7.1 In situ forming LLC gels

**Figure 5:**
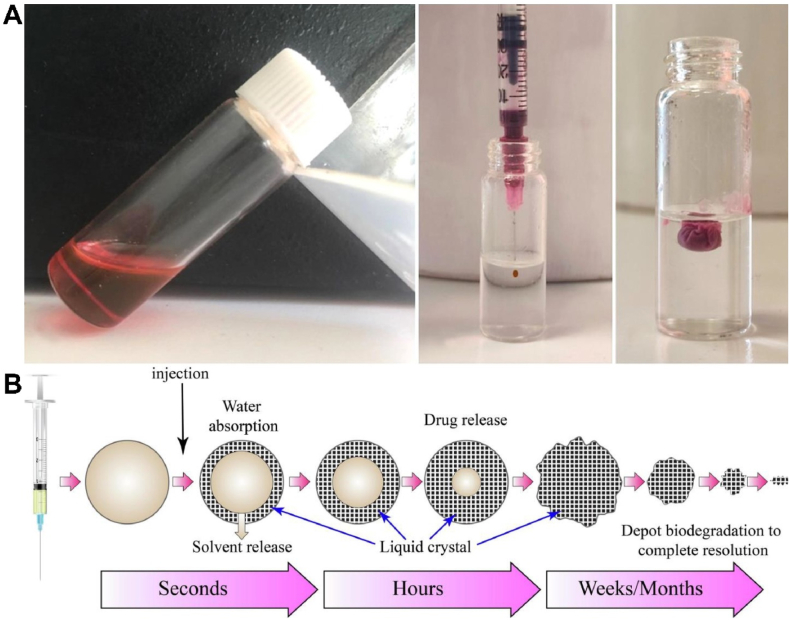
*In situ* forming LLC gels for long-acting therapy. (A) A precursor of doxorubicin LLC has good syringeability and transforms gel upon contacting water. Reprinted with permission from Ref. 115. Copyright © 2023 Elsevier. (B) Schematic illustration of LLC gel formation and long-acting therapy by controlled and sustained drug release. Reprinted with permission from Ref. 116. Copyright © 2022 Elsevier.

To address unmet clinical needs in long-acting drug delivery, *in situ* forming gels have emerged as promising alternatives, particularly those based on lipid systems capable of forming LLC gels. Compared to traditional polymeric systems, LLC-based gels offer several advantages, including superior biocompatibility, structural versatility, and the ability to provide stable, controlled, and sustained drug release over extended durations (Table 7 113, 114, 115, 116, 117, 118, 119, 120, 121, 122, 123, 124, 125). These systems are typically formulated by dissolving lipids in organic solvents such as ethanol or *N*-methyl-2-pyrrolidone to create a liquid precursor. Upon injection into an aqueous environment, rapid solvent exchange occurs: the solvent diffuses outward while water diffuses inward, triggering the spontaneous formation of a liquid crystalline gel (Fig. 5A)^115^. This transformation is nearly instantaneous upon contact with water (Fig. 5B)^116^. Studies have shown that water uptake reaches approximately 48.81% of the precursor weight within 5 min and exceeds 70% at equilibrium, which occurs around 2 h post-injection^114^. During this process, a portion of the drug is released rapidly in conjunction with the solvent exchange, while the remainder becomes embedded within the gel matrix and is released gradually over time as the matrix slowly dissolves. Clinically approved products such as Buvidal® and Posimir® utilize this *in situ* gel-forming technique for long-term analgesic applications.

**Table 7:** The advances of *in situ* forming LLC gels for long-acting therapy.

Drug	Disease	Material	Phase	Achievement	Ref.
Donepezil; cilostazol	Alzheimer’s disease	Phosphatidylcholine; *α*-tocopherol	H_II_	Compared with daily oral delivery, a single administration released for more than one month, not only elevating drug concentrations in brains, improving the clearing efficiency of brain macromolecules, reducing A*β* accumulation, enhancing cognitive functions of the aged mice, but improving patient compliance as well.	113
Rotigotine	Parkinson’s disease	Soybean phosphatidylcholine; GDO; ethanol	Q_II_	The precursor solution undergoes rapid exchange upon contact with PBS, and the diffusion of ethanol can reach 48.1% within 60 min and 80% within 8 h. The blood concentration remained above 0.1 ng/mL for 20 days and the bioavailability reached 72.59%. A 3.86-fold longer *t*_1/2_ and 16.43-fold higher AUC than solution.	114
Doxorubicin	Cancer	SMO; phosphatidyl choline; *N*-methyl-2-pyrrolidone; Tween 80	H_II_	A sustained release for 60 days. A significant decrease in tumor volume and enhancement of the survival rate compared to intravascular administration of solution. Prolonged accumulation at the tumor site, and a reduced systemic toxicity to the vital organs.	115
Naltrexone	Opioid and alcohol dependence	SMO; phosphatidylcholine; *N*-methyl-2-pyrrolidone	H_II_	Maintain the release of naltrexone for 30 days. *C*_max_ (ng/mL): 27.59 ± 2.48 (SMO), 27.63 ± 3.25 (GDO), 32.91 ± 2.21 (Vivitrol®); *T*_max_ (h): 36.00 ± 1.48 (SMO), 36.00 ± 1.73 (GDO), 48.00 ± 2.01 (Vivitrol®); MRT (h): 827.58 ± 23.46 (SMO), 672.73 ± 18.69 (GDO), 866.61 ± 25.62 (Vivitrol®); *t*_1/2_ (h): 561.31 ± 21.69 (SMO), 435.06 ± 15.86 (GDO), 583.53 ± 22.09 (Vivitrol®); AUC_0–t_ (ng.h/mL): 13609.63 ± 425.25 (SMO), 13218.28 ± 301.84 (GDO), 12790.633 ± 356.84 (Vivitrol®).	116
		GDO; phosphatidylcholine; *N*-methyl-2-pyrrolidone			
Diclofenac sodium	Pain	PT/ethanol/water: 76:19:5	Q_II_	The precursors transformed into gels in water. A 4.85-fold higher *t*_1/2_, 16-fold higher *T*_max_, 3.32-fold higher AUC, and 3.16 higher MRT than the solution.	117
		PT/ethanol/water/vitamine-E acetate: 72:18:5:5	H_II_	The precursors transformed into gels in water. A 3.4-fold higher *t*_1/2_, 8-fold higher *T*_max_, 2.97-fold higher AUC, and 2.67 higher MRT than the solution.	
Risperidone	Schizophrenia and bipolar disorder	Phospholipid S100/GDO; *N*-methyl-2-pyrrolidone (30%)	H_II_	A uniform and sustained serum levels for two months. The pharmacokinetic parameters in comparison with Risperdal CONSTA®: *C*_max_ (ng/mL), 47.31 ± 3.12 *vs.* 57.72 ± 4.24; *T*_max_ (h): 366 ± 4.18 *vs.* 720 ± 6.48; MRT (h), 850.51 ± 10.48 *vs.* 851.12 ± 7.34; *t*_1/2_ (h), 389.22 ± 7.22 *vs.* 246.39 ± 4.26; AUC_0–t_ (ng.h/mL), 57523.90 ± 49.76 *vs.* 46948.12 ± 48.54.	118
Doxorubicin	Murine 4T1 tumor mode	GMO; phosphatidylcholine; tocopherol acetate; d-*α*-tocopherol polyethylene glycol 1000 succinate; ethanol	L*α* to H_II_	The transformed depot system can be preprogrammed to provide tailored drug release intratumorally, over one week to one month. *In situ* formed LLC gels showed prolonged local and sustained drug release within tumor tissues, resulting in a 10-fold reduction in tumor volume and less cardiotoxicity compared with doxorubicin solution.	119
Doxorubicin; paclitaxel	Breast tumor	Phosphatidylcholine; GMO; tocopherol acetate; d-*α*-tocopherol polyethylene glycol 1000 succinate; ethanol	H_II_	Coaccommodate both hydrophobic and hydrophilic chemotherapeutic moieties. A low-viscosity injectable fluid with a lamellar phase that transforms into a hexagonal mesophase depot system upon intratumoral injection. The drug-loaded depot system locally provides sustained intratumoral delivery of the chemotherapeutics combination at their precisely synchronized ratio for over one month.	120
Kartogenin	Osteoarthritis	GMO; hyaluronic acid; *N*-methyl-2-pyrrolidone; water	Q_II_	Prolonged drug release and retention in the joint cavity to provide the combined effect of delivered drugs and hyaluronic acid.	121
Celecoxib					122
Minocycline hydrochloride	Periodontitis	PT; propylene glycol; water	Q_II_	A sustained release for four days; significantly reduced gingival index, probing depth, and alveolar bone loss.	123
Propolis flavonoids	Periodontitis	PT; carbitol; water	Q_II_	A sustained release for four days; Inhibited inflammatory cell infiltration, reactive oxygen species, and the expression of inflammatory cytokines. Alveolar bone and collagen were significantly regenerated	124
Aripiprazole	Schizophrenia and bipolar disorder	SMO; phosphatidylcholine; *N*-methyl-2-pyrrolidone	H_II_	A stable serum concentration and sustained release for about one month with a *T*_max_ of 120 h, a *t*_1/2_ of 458 h, and an MRT of 739 h, respectively.	125
		GDO; phosphatidylcholine; *N*-methyl-2-pyrrolidone	H_II_	A stable serum concentration and sustained release for about one month with a *T*_max_ of 144 h, a *t*_1/2_ of 841 h, and an MRT of 1190.57 h, respectively.	

AUC, area under the curve; *C*_max_, peak plasma concentrations; GDO, glycerol dioleate; H, hexagonal; L*α* lamellar; MRT, mean residence time; PT, phytantriol; Q, cubic; SMO, sorbitan monooleate; *t*_1/2_, half-life; *T*_max_, time to peak drug concentration.

Glycerol dioleate (GDO), phosphatidylcholine, sorbitan monooleate (SMO), and their combination are the main lipids to form the long-acting LLC gel, among which, the majority is H_II_ type (Table 7). Ethanol and *N*-methyl-2-pyrrolidone were the main solvents used to prepare the precursor solution (Table 7). Drug release from these systems can be sustained for one to two months, depending on formulation composition. Importantly, the type of liquid crystal formed can significantly influence pharmacokinetic behavior. For instance, PT-based LCs generally form cubic (Q_II_) phases but can be converted into hexagonal (H_II_) phases by incorporating additives such as vitamin E acetate^117^. In comparative studies, Q_II_-type formulations demonstrated longer pharmacokinetic profiles, including a twofold increase in time to peak concentration (16 h *vs.* 8 h), a 1.4-fold increase in half-life (11.21 h *vs.* 7.85 h), and a 1.2-fold increase in mean residence time (8.24 h *vs.* 6.96 h), despite showing similar *C*_max_ and AUC values to their H_II_-type counterparts.

### 7.2 Subcutaneous injection

**Figure 6:**
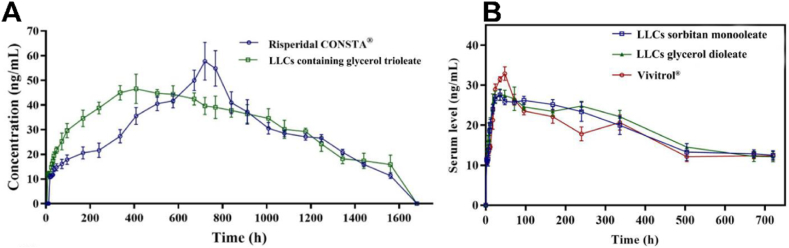
Comparison of pharmacokinetics between *in situ* forming LLC gels and approved long-acting preparations, (A) Risperdal CONSTA® and (B) Vivitrol®. Reprinted with permission from Refs. 118 and 116. Copyright © 2022 Elsevier and © 2022 Elsevier.

The *in situ* forming gel technique has been successfully applied *via* subcutaneous injection for pain management and the treatment of neurological disorders such as schizophrenia, Parkinson’s disease, Alzheimer’s disease, and opioid and alcohol dependence (Table 7). Optimized *in situ* forming LLC gels have demonstrated long-acting effects comparable to approved long-acting formulations. For example, an *in situ* forming LLC gel composed of phospholipid, GDO, and *N*-methyl-2-pyrrolidone could maintain uniform and sustained serum levels of risperidone for two months^118^. The gel exhibited a comparable AUC to Risperdal CONSTA®, poly lactic-*co*-glycolic acid (PLGA) microparticles of risperidone, but a lower peak concentration and a shorter peak time (Fig. 6A)^118^. Unlike PLGA microparticles, which release drugs *via* matrix degradation and show a prolonged lag phase, the LLC gel provided rapid attainment of effective plasma concentrations, potentially eliminating the need for supplemental oral antipsychotics often required with Risperdal CONSTA®. Similarly, *in situ* forming LLC gels composed of either GDO/phosphatidylcholine or SMO/phosphatidylcholine yielded pharmacokinetic profiles for naltrexone comparable to Vivitrol®, a PLGA microparticle preparation (Fig. 6B)^116^. Histological examination *via* hematoxylin–eosin staining revealed no signs of epidermal or dermal necrosis or inflammatory response induced by the gel, whereas the acidic microenvironment caused by PLGA erosion may provoke local inflammation. It is noteworthy that the physicochemical properties of the loaded drug influence the duration of release. Despite similar gel compositions, pharmacokinetic profiles differed between the lipophilic risperidone and the hydrophilic naltrexone. Risperidone blood levels were sustained for two months with a peak time of 366 h, whereas naltrexone concentrations were maintained for only one month with a peak time of 36 h^116^^,^^118^. This difference may be attributed to the stronger affinity of lipophilic drugs for the liquid crystalline matrix compared to hydrophilic compounds.

### 7.3 Intra-tumor, intra-articular, and periodontal administration

The lipid precursor can be administered *via* intratumoral injection to enhance drug accumulation at the tumor site while minimizing systemic toxicity. Using SMO and phosphatidylcholine as the matrix, doxorubicin release was sustained for up to 60 days^115^. The *in situ* forming LLC gel significantly reduced tumor volume and improved survival rates compared to intravenous administration of doxorubicin solution. The transformed depot can be preprogrammed for tailored intratumoral drug release ranging from one week to one month. *In situ* formed LLC crystal gels demonstrated prolonged local drug release within tumor tissues, achieving a tenfold reduction in tumor volume and reduced cardiotoxicity compared with doxorubicin solution^119^. Furthermore, the *in situ* forming LLC gel was able to co-encapsulate the lipophilic paclitaxel and hydrophilic doxorubicin at the tumor site, delivering synergistic effects^120^. Both drugs were released in a synchronized ratio for over one month. This combination therapy achieved a survival rate exceeding 66% by Day 60 without cardiotoxicity, enabling a reduction in total chemotherapeutic dose without compromising antitumor efficacy.

For osteoarthritis and cartilage defect treatment, a precursor composed of GMO and hyaluronic acid was developed for intra-articular administration of kartogenin or celecoxib^121^^,^^122^. The resulting *in situ* formed cubic gels exhibited high stiffness, which helped attenuate joint movement stress on subchondral bone and provided structural support for cell proliferation and differentiation. Additionally, the gels demonstrated excellent lubrication to reduce cartilage abrasion from joint friction. Importantly, drug release and retention in the joint cavity were sustained for over four weeks, facilitating a combined therapeutic effect of the drugs and hyaluronic acid.

*In situ*-formed liquid crystal gels are also advantageous for administration into periodontal pockets to treat periodontitis. A precursor composed of PT, propylene glycol, and water rapidly formed a cubic phase gel within 7 s upon exposure to excess water, providing sustained release of minocycline hydrochloride for four days^123^. Alternatively, formulations containing PT, carbitol, and water produced thermosensitive hydrogel depots that transitioned to a cubic semisolid state at 37 °C^124^. These gels remained stable in the periodontal pocket and sustained the release of propolis flavonoids for four days. Both systems significantly reduced gingival inflammation, probing depth, and alveolar bone loss.

## 8 Concluding remarks

LLCs have demonstrated immense potential as versatile drug delivery systems, offering solutions to some of the most pressing challenges in biomedicine. Their ability to encapsulate both hydrophilic and hydrophobic drugs, enhance solubility, and provide controlled and sustained release makes them highly valuable for preparation development, overcoming biological/biofilm barriers, enhancing bioavailability, achieving long-acting effects, promoting wound healing, and site-specific delivery. Advances in LLC formulations, such as nanoparticles, gels, and *in situ* forming systems, enable diverse functionalities following different administration routes. For example, LCNPs have shown promising results in overcoming the stratum corneum for transdermal drug delivery, enhancing transcorneal permeation in ocular applications, and bypassing the blood–brain barrier *via* intranasal administration. Surface modification and incorporation of stimuli-responsive properties reduce the off-target effect of LCNPs. Furthermore, *in situ* forming LLC gels have revolutionized localized drug delivery, offering sustained drug release for chronic conditions such as osteoarthritis, cancer, and opioid dependence. With ongoing research and development, LLC-based systems have the potential to significantly improve the management of various diseases by offering more efficient and less invasive treatment options.

Despite the advances, challenges remain in fully realizing the potential of LLC-based drug delivery systems. The constituents forming LLCs are limited, mainly focusing on GMO and PT. It is essential to expand the material library to improve the properties of the systems and expand their biomedical applications. The analogs of GMO and the combination of lipid materials, such as phospholipids and fatty acids, provide a solution. In addition, the combination with functional materials, such as permeation enhancers, PEGylated phospholipids, and pH-sensitive materials, may offer enhanced drug stability, improved permeability, better control over release kinetics, and improved targeting.

Despite their structural advantages, LLCs face significant route-specific limitations. Oral delivery is hindered by pH/enzyme-driven instability and food effects; parenteral routes suffer from high viscosity (requiring large needles) and sterilization challenges; topical applications struggle with poor skin penetration and residual tackiness; ocular use risks blurred vision and short retention. Universal constraints include low drug loading for macromolecules, manufacturing complexity, oxidative instability of lipid components, high material costs, and unpredictable *in vivo* phase transitions, collectively impeding clinical translation despite their potential for controlled release.

LLCs designed for different administration routes share fundamental principles but exhibit route-specific priorities. The common design strategy involves selecting suitable amphiphiles to form target mesophases with sufficient drug-loading capacity to meet therapeutic dosage requirements. Divergences arise from distinct biological challenges across routes. For oral delivery, the focus lies on maintaining drug solubilization and enhancing absorption. Lipid/mesophase selection must prioritize bile salt/enzyme resistance, mucoadhesion, and efficient intestinal epithelial transport. Intravenous formulations require optimization for long circulation, targeting capability, and colloidal stability. Transdermal, ocular, and nasal LLCs emphasize bioadhesion at the administration site and disruption of biological barriers. Local implants aim for sustained release, necessitating high-viscosity matrices with prolonged release properties.

The mesophase may affect the efficacy of drugs. Bicontinuous cubic phases (*e.g.*, Ia3d/Pn3m) enhance efficacy *via* sustained release from intertwined aqueous/lipid channels, maintaining therapeutic plasma levels while their high interfacial curvature fuses with biological membranes (*e.g.*, enhancing transdermal flux *via* SC lipid disruption); Hexagonal phases, with cylindrical aqueous cores surrounded by lipids, enable anisotropic biphasic release—rapid hydrophilic drug burst for acute conditions (*e.g.*, wound infection) followed by sustained diffusion, leveraging high viscosity for mucoadhesion; Lamellar phases, characterized by planar lipid bilayers, provide gradual diffusion-controlled release ideal for stabilizing biologics but exhibit minimal membrane perturbation, limiting barrier penetration while excelling as non-irritating depots. Critically, *in vivo* phase transitions dynamically modulate drug exposure, necessitating geometry-specific design to match pathological barriers and pharmacokinetic requirements.

LCNPs offer superior drug-loading versatility (hydrophobic/amphiphilic/hydrophilic drugs), enhanced bio-barrier penetration (*e.g.*, skin, BBB), and tunable sustained release *via* phase transitions, outperforming liposomes (limited by bilayer partitioning and burst release) and polymeric micelles (dilution-triggered dissociation, primarily hydrophobic loading). However, LCNPs face complex formulation/scaling challenges, scarce clinical data, and rigorous characterization needs, unlike well-established liposomes or polymer-flexible micelles. Thus, LLCs excel in precision applications like barrier traversal or long-acting depots but require greater nanoengineering expertise, while liposomes/micelles remain pragmatic choices for simpler delivery scenarios.

The integration of LLCs into mainstream pharmaceutical formulations could pave the way for more efficient, patient-friendly, and targeted therapies, marking a new era in drug delivery innovation. The bulk LLCs exhibit the properties of hydrogel and can be administered similarly. Attention should be paid to the loading of drugs and incorporation of other excipients, as they may impair the drug delivery capacity by damaging the structures. Diverse approaches have been established to prepare LCNPs. The stability may be an obstacle to their clinical translation. Although P407 can stabilize the dispersed LCNPs, the long-term stability is yet to be explored. Alteration in temperature and water content may induce the change of mesophase, leading to drug leakage, size growth, and other instability issues. In addition, it is impossible to transfer LCNP suspensions to solid precursors *via* lyophilization or mixing with solid-state adsorbents, which have been widely applied to other nanoparticles like micelles and polymeric nanoparticles. The formation of LLCs cannot be separated from the presence of water. These solidification processes destroy the structure of LLCs. Ongoing research should focus on alternative solutions. The *in situ* forming LLC gels are ease in application and have been approved. However, the organic solvent induces severe pain upon subcutaneous injection and may induce local or systemic toxicity. Phase transition induced by water absorption or other stimuli that have been adopted in intranasal and intravenous administration may be a good approach to avoid the use of organic solutions.

The mechanisms for the enhanced permeability from LLCs across biological barriers are yet to be unraveled. DSC and FTIR studies confirmed the disrupting effects of LLCs on the biological barriers. Our knowledge of how this effect occurs and the influencing factors is limited. Answers to these issues help optimize the composition and types of LLCs. Fluorescence labeling has been widely adopted to illustrate the permeation-enhancing effects of LCNPs. The results didn’t support the penetration of the intact nanoparticles as the fluorophore is not environment-responsive. The observed signals represent both the free fluorophores and the nanoparticles. Only the environment-responsive fluorophores can lead to a sound conclusion, which facilitates refining the design of LCNPs to achieve more efficient drug delivery.

Future research should focus on optimizing the stability, safety, and efficacy of LLC-based formulations, alongside advancing scalable manufacturing techniques. Bridging the gap between laboratory innovation and clinical application will enable LLCs to serve as a cornerstone for next-generation biomedical technologies.

## Author contribution

Guojin Liu: Writing – original draft, Visualization. Yuanmei Yang: Writing – original draft. Qing Wu: Writing – original draft. Zhongjian Chen: Supervision, Visualization. Nadia M. Hamdy: Writing – review & editing. Amr Amin: Writing – review & editing. Gang Chen: Writing – review & editing, Supervision. Yi Lu: Writing – review & editing, Supervision, Conceptualization. All of the authors have read and approved the final manuscript.

## Conflicts of interest

The authors have no conflicts of interest to declare.
